# A MEMS Microbolometer-Based ATR Mid-Infrared Sensor for Direct Dissolved CO_2_ Detection and UV-Induced Sediment Carbon Assay in Aquatic Environments

**DOI:** 10.3390/s26092689

**Published:** 2026-04-26

**Authors:** Md. Rabiul Hasan, Amirali Nikeghbal, Steven Tran, Farhan Sadik Sium, Seungbeom Noh, Hanseup Kim, Carlos H. Mastrangelo

**Affiliations:** Department of Electrical and Computer Engineering, University of Utah, Salt Lake City, UT 84112, USA; mdrabiul.hasan@utah.edu (M.R.H.); amirali.nikeghbal@utah.edu (A.N.); steven.tran@utah.edu (S.T.); farhan.sium@utah.edu (F.S.S.); moses.noh@gmail.com (S.N.); hanseup.kim@utah.edu (H.K.)

**Keywords:** microbolometer, ATR spectroscopy, dissolved CO_2_ sensor, UV photo-oxidation, sediment organic carbon, in situ monitoring, evanescent wave, mid-infrared detection, MEMS sensor, infrared sensor, aquatic carbon cycling

## Abstract

**Highlights:**

- A novel low-cost microbolometer-based ATR mid-infrared sensor achieves linear dissolved CO_2_ detection (R^2^ ≈ 0.99) from ~50 to 1000 ppm with LOD ~25 ppm at lower temperatures (5–10 °C).
- UV-C irradiation (254 nm) induces the highest net CO_2_ production from soil-water mixtures (ΔCO_2_ ≈ 339 ppm in 30 min), far exceeding UV-B and UV-A, due to direct photolytic cleavage of organic bonds.

**The implications of the main findings**

- The sensor enables reliable in situ monitoring of dissolved CO_2_ across environmental gradients (temperature 5–35 °C, pH 5–11, pressure 1–1.5 ATM) with >90% accuracy versus commercial reference instruments.
- Combined real-time CO_2_ detection and rapid UV-stimulated SOC assay capability offers a versatile, field-deployable platform for aquatic carbon cycling and sediment organic carbon studies.

**Abstract:**

Monitoring dissolved carbon dioxide (CO_2_) in aquatic and sediment systems is critical for understanding carbon cycling and climate feedback. This study develops and characterizes a compact, low-cost microbolometer-based attenuated total reflectance (ATR) mid-infrared sensor for direct dissolved CO_2_ measurement in liquid and soil-water environments. The system integrates a ZnSe ATR crystal with custom suspended SiN membrane microbolometers and uses evanescent-wave absorption at 4.26 μm with a broadband LED source and computational spectral reconstruction, eliminating the need for an interferometer. Calibration shows excellent linearity (R^2^ ≈ 0.99) over 50–1000 ppm CO_2_, with a practical limit of detection (LOD) of ~26–35 ppm at 5–25 °C. UV-induced CO_2_ generation from soil-water mixtures was investigated across UV wavelengths, revealing UV-C (254 nm) as optimal, producing net ΔCO_2_ ≈ 339 ppm above ambient levels in 30 min. Environmental factors (temperature 5–35 °C, pH 5–11, pressure 1–1.5 ATM, dissolved organic carbon) were systematically evaluated, confirming robust sensor performance (accuracy >90%, correlation r > 0.98 with reference instrument). This sensor represents the first integration of MEMS microbolometer detectors with ATR evanescent-wave spectroscopy for liquid-phase dissolved CO_2_, enabling real-time monitoring and rapid sediment organic carbon assessment in a field-deployable platform.

## 1. Introduction

Carbon dioxide (CO_2_) is a critical greenhouse gas whose dynamics in aquatic and sediment environments are central to global carbon cycling and climate change research [1,2,3]. Ocean sediments, freshwater systems, and soils collectively represent major carbon reservoirs, where complex interactions among organic matter decomposition, microbial respiration, photochemical oxidation, and environmental factors govern CO_2_ fluxes [4,5]. A comprehensive understanding of these processes requires accurate, real-time, and in situ CO_2_ detection technologies capable of operating under challenging underwater conditions, including elevated hydrostatic pressure, low temperatures, light attenuation, and corrosive environments [6,7].

The significance of dissolved CO_2_ monitoring extends across multiple domains. In oceanography, dissolved CO_2_ (pCO_2_) measurements are essential for studying ocean acidification, a process driven by the absorption of anthropogenic CO_2_ by seawater, which lowers pH and threatens marine ecosystems [8,9]. The Global Carbon Budget 2024 reports total anthropogenic CO_2_ emissions of 11.1 ± 0.9 GtC yr^−1^, with the ocean absorbing 2.9 ± 0.4 GtC yr^−1^, approximately 26% of total emissions [2]. In typical seawater at pH 8.1, over 90% of dissolved inorganic carbon exists as bicarbonate (HCO_3_^−^) or carbonate (CO_3_^2−^), with less than 1% remaining as free dissolved CO_2_ [10]. This carbonate buffering system means that small changes in pH, temperature, or pressure can greatly influence how much CO_2_ is released or retained. In sediment science, quantifying soil organic carbon (SOC) turnover is critical for carbon stock assessments and for predicting how climate warming may accelerate carbon release from permafrost and coastal sediments [11,12,13]. Recent studies have shown that UV-driven photochemical mineralization of dissolved organic carbon (DOC) to CO_2_ in surface waters constitutes a significant fraction of the Arctic carbon budget [14]. Marine sediments contain an estimated 1750 GtC in the upper layers [3], and benthic carbon fluxes in coastal sediments range from 10 to over 100 mmol CO_2_ m^−2^ d^−1^ depending on organic loading, temperature, and oxygen availability [15]. Understanding these processes requires sensors that can operate continuously and autonomously in remote or harsh environments.

### 1.1. State-of-the-Art Underwater CO_2_ Sensing Technologies

Several commercial and laboratory-scale technologies currently exist for underwater CO_2_ detection. Non-dispersive infrared (NDIR) sensors are the most widely deployed technology for atmospheric CO_2_ monitoring, offering good specificity and sensitivity by exploiting the strong 4.26 μm absorption band of CO_2_ [16]. However, adapting NDIR for underwater use is inherently challenging because infrared absorption measurements typically require a gas-phase sample or an equilibrated headspace. Commercial submersible pCO_2_ sensors, such as the Pro-Oceanus CO_2_-Pro CV ($15,000–$20,000), the CONTROS HydroC ($20,000+), and the Sunburst Sensors SAMI-CO_2_ rely on membrane-equilibrated gas cells or optical chemical indicators to measure dissolved CO_2_ partial pressure [17,18,19]. While these instruments achieve high accuracy (±2 μatm typical), they are bulky (often >30 cm in length), expensive, power-hungry (>1 W continuous), and slow to equilibrate (response times of 30–60 s), limiting their use in distributed sensor networks or on miniaturized platforms such as autonomous underwater vehicles (AUVs) or micro-ROVs [20].

Other CO_2_ sensing technologies, such as Electrochemical CO_2_ sensors based on Severinghaus-type electrodes, offer an alternative but suffer from drift, cross-sensitivity, and limited lifetime under harsh marine conditions [21]. Another technology includes optical fiber-based sensors using fluorescence indicators which have shown promise for pH and pCO_2_ measurements but are susceptible to photobleaching and biofouling [22]. More recently, mid-infrared laser-based spectroscopy systems have been proposed for high-precision dissolved carbon measurements, such as the DOE-funded SLEUTH project using towed laser-optical sensors [23], but these remain in early research stages and are not yet miniaturized for routine deployment. Schädle et al. (2016) developed a portable IR-ATR system using a diamond internal reflection element for dissolved CO_2_ and CH_4_ monitoring in brine at pressures up to 11 MPa [24,25], while Li et al. (2022) demonstrated microscale IR spectroscopy for dissolved CO_2_(aq) quantification up to 58 atm [26]. However, these systems utilize bench-top FTIR spectrometers with cryogenically cooled MCT detectors, impractical for autonomous field deployment.

Liu et al. (2023) comprehensively reviewed recent advances in marine dissolved gas sensing, noting the persistent gap between laboratory-grade spectroscopic techniques and field-deployable instruments [6]. A common limitation of all the above approaches is that none provides a low-cost, compact, low-power solution that can directly measure dissolved CO_2_ in liquid or sediment phases without requiring membrane equilibration, headspace extraction, or cryogenic cooling. This gap motivates the development of new sensor architectures based on compact uncooled infrared detector platforms such as microbolometers [27].

### 1.2. ATR Spectroscopy for Aquatic Measurements

**Figure 1:**
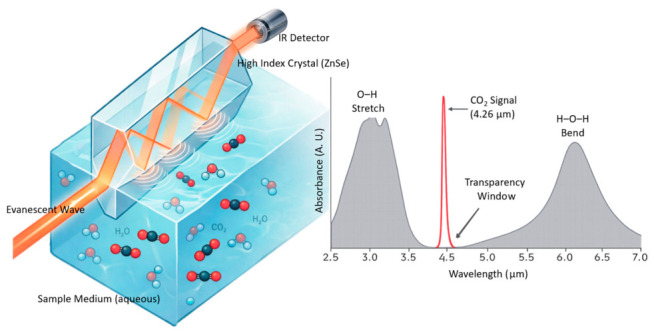
Evanescent wave penetration and interface dynamics in ATR-Based CO_2_ sensing. This schematic was generated with AI assistance (ChatGPT 5.2, University of Utah Workspace) and reviewed and edited by the authors.

Attenuated Total Reflectance (ATR) infrared spectroscopy is a well-established analytical technique that enables direct chemical analysis of liquids, solids, and semi-solid samples by exploiting the evanescent wave generated at the interface of an infrared-transparent crystal [28]. When an IR beam undergoes total internal reflection inside a high-refractive-index crystal (such as zinc selenide, diamond, or germanium), an evanescent wave penetrates a few micrometers into the adjacent medium, interacting with molecular species at or near the surface as shown in Figure 1. The penetration depth dp is given by:

dp = λ/[2πn_1_(sin^2^θ − (*n*_2_/*n*_1_)^2^)^(0.5)] (1)

where λ is the wavelength, n_1_ and n_2_ are the refractive indices of the crystal and medium, and θ is the angle of incidence [28]. For our ZnSe crystal (*n*_1_ = 2.4) at λ = 4.26 μm and θ ≈ 45°, dp ≈ 1.0–1.5 μm. This surface-sensitive technique is particularly advantageous for aqueous measurements because the evanescent wave probes only a thin layer, reducing the overwhelming absorption of bulk water while retaining sensitivity to dissolved species.

A key advantage of the ATR configuration for dissolved CO_2_ measurement is spectral selectivity. Dissolved CO_2_(aq) retains the same fundamental ν_3_ asymmetric stretch near 2349 cm^−1^ (4.26 μm) as gas-phase CO_2_, broadening slightly (5–10 cm^−1^) due to hydrogen bonding with the water solvent cage [24,26]. Critically, dissolved CO_2_(aq) is spectrally distinct from the carbonate species: bicarbonate absorbs at 1360 and 1010 cm^−1^, while carbonate absorbs at 1415 and 880 cm^−1^, well separated from the 4.26 μm CO_2_ band [24]. Furthermore, water’s mid-IR absorption leaves a relative transparency window around 4.0–4.5 μm (between the O-H stretch at ~3.0 μm and the H-O-H bend at ~6.1 μm), through which the CO_2_ ν_3_ band can be interrogated with minimal interference. The shallow evanescent penetration depth (~1.0–1.5 μm) further reduces the effective water absorption compared to transmission cells.

Combining ATR spectroscopy with compact infrared detectors such as microbolometers represents a promising pathway toward miniaturized, low-power CO_2_ sensors. Microbolometers are uncooled thermal IR detectors that measure temperature changes caused by absorbed IR radiation through resistance variations in a suspended membrane [27,29,30]. They operate without cryogenic cooling, consume milliwatts of power, and can be fabricated at low-cost using MEMS processes. Previous work by Barritault et al. [31] demonstrated microbolometer-based NDIR CO_2_ sensors with ppm-level sensitivity at only 1.35 mJ per measurement. Dubey et al. (2024) evaluated low-cost NDIR CO_2_ sensors and proposed machine learning calibration to improve accuracy to ±5 ppm [16]. Zhang et al. (2023) developed compact NDIR configurations with optimized conical chambers for portable gas analysis [32]. Our research group has further advanced microbolometer technology with suspended SiN membrane designs achieving responsivities of 43,000–48,000 V/W [29,33], enabling high-sensitivity mid-IR detection without vacuum packaging.

### 1.3. UV-Induced Photochemical CO_2_ Generation

Beyond passive detection of existing CO_2_, there is growing scientific interest in using ultraviolet light to actively stimulate CO_2_ generation from organic matter in soils and sediments. UV-induced photochemical oxidation is a well-documented pathway for mineralizing dissolved organic carbon (DOC) to CO_2_ in natural waters [11,12,13,14]. Recent studies by Doane (2025) demonstrated that photochemical CO_2_ emission from soil is nearly ubiquitous across diverse environments and comparable in magnitude to microbial respiration, yet this source remains largely unrepresented in carbon cycle models [4]. Cory et al. (2013, 2017) showed that surface exposure of permafrost soil carbon to sunlight stimulates substantial CO_2_ release, with photochemical processing supplying approximately one-third of CO_2_ released from Alaskan arctic surface waters [12,13]. Ward and Cory (2016) further demonstrated that partial photo-oxidation of DOC shifts microbial metabolic pathways and stimulates subsequent biological respiration [11]. Grasset et al. (2024) established that DOM photo reactivity decreases with increasing water residence time as chromophoric compounds are progressively depleted [14].

The mechanism involves UV photons generating reactive oxygen species (ROS), including singlet oxygen (^1^O_2_), superoxide radicals (O_2_⋅^−^), and hydroxyl radicals (⋅OH), which oxidize organic molecules [34,35]. UV-C photons (>4.4 eV) can directly cleave C-C and C-O bonds (dissociation energies 3.6 and 3.7 eV, respectively), while UV-A/B generates ROS indirectly through photosensitization of chromophoric dissolved organic matter (CDOM) [36].

This UV-induced approach offers a novel strategy for in situ quantification of soil organic carbon: by shining UV on a sediment or soil-water mixture and measuring the resulting CO_2_ produced, one can rapidly estimate the labile organic carbon content without lengthy chemical digestion or combustion [37]. The PeCOD analyzer uses a related photocatalytic oxidation principle for SOC estimation [38] but lacks the spectroscopic specificity and portability of our approach. Our group previously demonstrated a UV-based, in situ, low-power wireless soil carbon measurement system that generated up to 128 ppm of CO_2_ from soil within 30 min of UV exposure [37].

### 1.4. Objectives and Novelty of This Work

This paper presents a comprehensive study that integrates microbolometer-based ATR mid-infrared absorption spectroscopy with UV-induced CO_2_ generation for dual-function environmental sensing. To the best of our knowledge, this work represents the first reported use of a MEMS suspended SiN membrane microbolometer coupled with ATR evanescent wave spectroscopy for direct liquid-phase dissolved CO_2_ detection. While microbolometers have been widely employed for thermal imaging and gas-phase NDIR sensing [16,32], ATR spectroscopy for dissolved CO_2_ has previously been implemented mainly with laboratory infrared systems [24,25,26]. No prior work has combined these two technologies for direct aqueous-phase CO_2_ measurement, eliminating the membrane equilibration step that limits response time and adds complexity to existing commercial underwater CO_2_ sensors.

Beyond sensor development, this study systematically investigates UV-induced photochemical CO_2_ generation from soil-water mixtures under UV-A, UV-B, and UV-C irradiation, identifying UV-C as the most effective wavelength band for rapid CO_2_ production and providing a mechanistic explanation for the observed wavelength dependence. The effects of key environmental parameters like pH (7–10), temperature (5–35 °C), pressure (1–1.5 ATM), and organic compost loading on UV-induced CO_2_ production are comprehensively characterized under simulated subsurface conditions. Sensor accuracy is validated against a commercial reference instrument, with the system demonstrating greater than 90% reliability across all tested environmental ranges. Notably, combining ATR-based CO_2_ detection with UV stimulation enables a dual-function platform capable of both real-time dissolved CO_2_ monitoring and rapid sediment organic carbon (SOC) assay, a capability unavailable in any existing commercial system. The following sections describe the sensor system design and experimental methods (Section 2), present calibration, UV-induced CO_2_ generation, and environmental factor results (Section 3), discuss implications and comparisons (Section 4), and conclude with future directions (Section 5).

## 2. Materials and Methods

This section describes the sensor system architecture and its underlying detection principle (Section 2.1), followed by the calibration procedure and low-temperature testing protocol (Section 2.2), the UV-induced CO_2_ production experiments (Section 2.3).

### 2.1. Microbolometer ATR CO_2_ Sensor System Design

**Figure 2:**
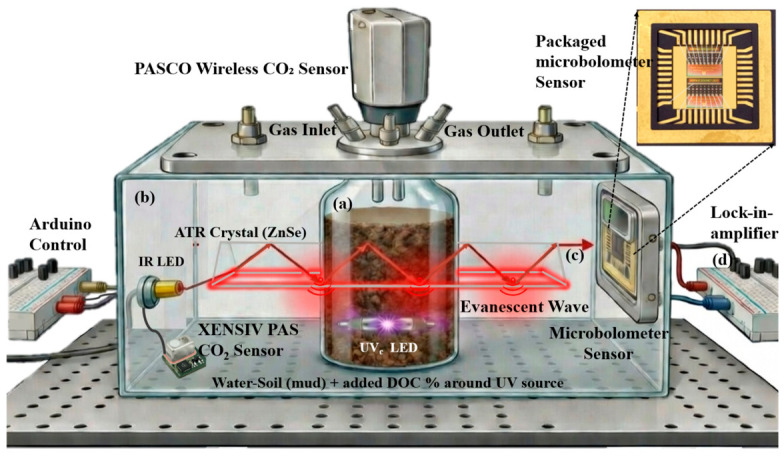
Schematic diagram of the dual-chamber microbolometer-based ATR CO_2_ detection system showing: (**a**) inner chamber containing aqueous sample with immersed ZnSe ATR crystal and gas inlets/outlets for N_2_ and CO_2_; (**b**) sealed inner chamber with commercial PASCO CO_2_ reference sensor [39]; (**c**) optical path from Hamamatsu LED IR source (L15895) through ATR crystal to suspended SiN membrane microbolometer array; (**d**) lock-in amplifier and data acquisition system.

The CO_2_ detection system is built around an Attenuated Total Reflectance (ATR) configuration with a MEMS microbolometer array as the infrared detector. The sensor employs a dual-chamber architecture specifically designed to enable precise dissolved CO_2_ quantification while protecting the fragile microbolometer from direct water contact. A schematic diagram of the complete system is shown in Figure 2.

#### 2.1.1. Dual-Chamber Design

The suspended silicon nitride membrane at the core of the microbolometer is electrically and mechanically fragile; direct immersion in water would cause short circuits and irreversible damage [30]. To overcome this constraint while still enabling liquid-phase measurements, the sensor employs a dual-chamber design that physically separates the aqueous sample from the detector while coupling them optically through the ATR crystal.

The inner chamber (~50 mL volume) contains the aqueous sample, either deionized water or a sediment-water mixture, with the ZnSe ATR crystal (25 mm × 10 mm × 2 mm, 45° facets, refractive index, *n* = 2.4 at 4.26 μm) fully immersed. Gas inlets for nitrogen (N_2_) and carbon dioxide (CO_2_) allow controlled introduction of calibration gases, while an outlet maintains regulated gas flow. A commercial PASCO wireless CO_2_ sensor (PS-3208, detection range 0–100,000 ppm, accuracy ±25 ppm + 5% of reading) is also housed within this chamber to provide continuous reference measurements for cross-validation.

Surrounding this inner chamber, the sealed outer chamber is purged with pure N_2_ through dedicated inlet and outlet ports, ensuring that no ambient CO_2_ contaminates the optical path between the ATR crystal exit face and the microbolometer detector. An XENSIV PAS CO_2_ sensor (Infineon Technologies, Neubiberg, Germany) was additionally placed in the outer chamber for real-time atmospheric CO_2_ monitoring to verify N_2_ purge effectiveness, maintaining a stable and CO_2_-free baseline in the detection path.

#### 2.1.2. Optical and Electronic Components

Having described the physical architecture that separates the aqueous sample from the detector, we now detail the optical and electronic subsystems that perform the actual infrared measurement. A key distinction must be made at the outset: unlike conventional Fourier Transform Infrared (FTIR) spectroscopy, which employs a broadband source combined with a Michelson interferometer to encode and reconstruct spectral information via the Fourier transform [28], our system achieves spectral selectivity through a fundamentally different approach, computational mid-infrared spectrometry. An array of wavelength-selective microbolometers, each with a distinct spectral absorption response, is illuminated by a broadband infrared source, and the sample’s absorption spectrum is computationally reconstructed from the differential detector responses via a regularization algorithm [29,30]. No interferometer, moving mirror, or Fourier transform processing is involved. This approach preserves the ability to resolve the CO_2_ absorption at 4.26 μm while dramatically reducing system complexity, size, and cost compared to bench-top FTIR instruments [40].

The broadband infrared source used is a Hamamatsu LED (model L15895) from Boston Electronics, Brookline, MA in USA with emission spanning approximately 3–5 μm, encompassing the CO_2_ ν_3_ absorption band at 4.26 μm. The LED is electrically modulated with an Arduino controller at 37 Hz with a 50% duty cycle to enable lock-in detection. On the detection side, the microbolometer array consists of custom-fabricated suspended SiN membrane elements with wavelength-skewed spectral responses across the 3–5 μm range [29,30]. Each element features a suspended silicon nitride platform with a lightly doped polysilicon thermistor for resistance-based temperature readout, and an infrared absorber layer positioned above a platinum mirror. Together, the absorber (top) and platinum mirror (bottom) form a Fabry-Pérot optical cavity, with the SiN dielectric layer serving as the resonant spacer. By varying the spacer thickness across array elements during microfabrication, each element’s peak absorption wavelength is tuned to a different position within the 3–5 µm band, producing the wavelength-selective response functions that enable computational spectral reconstruction [29,30]. The suspended membrane architecture provides thermal isolation from the substrate, achieving responsivities of R_v_ = 43,000–48,000 V/W, approximately a 10-fold increase over substrate-based microbolometers [30,33]. Additional device performance parameters, characterized in our previous work, include noise equivalent power NEP ≈ 0.2–0.5 nW/√Hz and specific detectivity D ≈ 1–2 × 10^8^ cm·√Hz/W, measured at atmospheric pressure without vacuum packaging [29,30,33].

The signal from each microbolometer element is demodulated using a lock-in amplifier (Stanford Research SR830) referenced to the 37 Hz LED modulation frequency. The lock-in time constant was set to 3 ms, matched to the intrinsic thermal time constant of the suspended SiN microbolometer elements (~4.3 ms) [29,33]. This thermal time constant is longer than the microsecond to sub-millisecond values reported for commercial imaging microbolometers, which employ small pixel plates (17–25 μm pitch) optimized for fast frame rates. Our devices are instead designed as large-area single-element spectral detectors with suspended plates ≥500 μm on a side and long, thin support beams, deliberately maximizing responsivity through increased thermal mass and reduced thermal conductance, which shifts τ = C/G into the millisecond range. Atmospheric-pressure operation contributes additional air-gap heat loss but eliminates hermetic wafer-level packaging requirements. The matched lock-in and detector time constants provide optimal signal extraction while rejecting out-of-band noise. At the system level, the measurement stabilization time (~3 s) is limited by CO_2_ diffusion and equilibration within the evanescent wave sensing zone, not by the detector or readout electronics.

This phase-sensitive detection extracts the modulated CO_2_ absorption signal while rejecting 1/f noise, ambient thermal drift, and the static water absorption background, achieving noise floors of 0.15–0.2 μV. For each measurement, the lock-in demodulated signals from the individual microbolometer array elements were processed through the computational spectral reconstruction algorithm described in [29,30] to recover the sample’s mid-infrared absorption spectrum across the 3–5 μm band. The reconstructed absorbance intensity at the CO_2_ ν_3_ band (4.26 μm, 2349 cm^−1^) was extracted as the primary analytical signal. Throughout this work, this reconstructed CO_2_ band intensity is reported as an equivalent voltage (μV) referenced to the lock-in amplifier output scale, as it is this quantity that is directly measured and calibrated. The voltage-to-concentration conversion is established through the calibration procedure described in Section 2.2. The combination of broadband illumination, wavelength-selective detection, computational spectral reconstruction, and lock-in demodulation constitutes the complete signal chain from dissolved CO_2_ molecules to a quantitative voltage output.

#### 2.1.3. Detection Principle

The hardware described above exploits the evanescent wave formed when infrared light undergoes total internal reflection inside the ZnSe ATR crystal. When infrared light propagates through the ZnSe ATR crystal and strikes the crystal-sample interface at an angle exceeding the critical angle, total internal reflection occurs, generating an evanescent wave that extends a short distance into the aqueous sample [24]. The evanescent wave penetrates approximately 1.0–1.5 μm into the aqueous sample (Equation (1)), where dissolved CO_2_(aq) absorbs IR radiation at its characteristic ν_3_ asymmetric stretching band near 4.26 μm (2349 cm^−1^) [24,26]. The relationship between absorbance and dissolved CO_2_ concentration follows a modified Beer-Lambert law adapted for the multi-reflection ATR geometry:

A = ε · C · N · dp(2)

where ε is the molar absorptivity coefficient (L mol^−1^ cm^−1^), C is the dissolved CO_2_(aq) concentration (mol/L), N is the number of internal reflections at the sample-contacting surface, and d_p_ is the penetration depth per reflection from Equation (1). For our ZnSe crystal (L = 25 mm, t = 2 mm, facet angle θ = 45°), the geometric maximum number of reflections is N_max_ = L/(2t·tanθ) ≈ 6. In practice, the effective N is reduced to approximately 3–4 because the MIR-LED source is non-collimated (beam divergence ~±15° half-angle), causing rays to enter the crystal at a distribution of angles and undergo varying numbers of reflections; additionally, the finite beam diameter (~3 mm) and Fresnel coupling losses at the facets reduce the contribution of marginal rays. The product N · d_p_ represents the total effective optical path through the sample (~4–6 μm). Because absorbance scales linearly with concentration for moderate values, the microbolometer output voltage change is directly proportional to dissolved CO_2_ concentration. Importantly, the empirical calibration procedure (Section 2.2) captures the effective product N · d_p_ · ε as a single lumped parameter within the measured sensitivity (0.017 µV/ppm), making the quantitative CO_2_ determination independent of the precise value of N.

Since the broadband LED illuminates the full 3–5 μm range, the wavelength-selective microbolometer array captures differential absorption across the entire band. The spectral reconstruction recovers the sample’s mid-infrared absorption spectrum from the array response vector using a regularized inversion. Each microbolometer element produces an output r_n_ = ∫ f_n_(λ) x(λ) dλ, where f_n_ (λ) is the element’s spectral response function and x(λ) are the incident spectrum. In matrix form, the response vector Y = R·X, where R is the responsivity matrix formed by the calibrated spectral responses of all array elements. Direct inversion of R is ill-conditioned because the broad, overlapping filter responses yield a Fredholm integral equation of the first kind, which produces non-physical oscillatory solutions under naive inversion [29]. This is resolved through regularization methods that constrain the recovered spectrum to physically meaningful solutions. The CO_2_ ν_3_ absorption band at 4.26 µm is spectrally well separated from water absorption features and from potential interferents such as dissolved CH_4_ (3.3 µm) and H_2_O bending (6.1 µm), enabling reliable isolation of the CO_2_ signal even in the presence of complex aqueous-phase background absorptions. A detailed derivation and validation of the reconstruction algorithm is provided in [29,30]. The reconstructed absorbance at this wavelength serves as the quantitative analytical signal, reported as an equivalent voltage (μV) throughout this work. Simultaneously, the lock-in demodulation rejects static water absorption and ambient thermal background. This combined approach enables selective dissolved CO_2_ quantification despite the presence of strong water absorption in the mid-infrared region.

### 2.2. Calibration and Low-Temperature Testing Protocol

With the detection principle established, a reliable calibration procedure is needed to convert the microbolometer voltage output into dissolved CO_2_ concentration. This is achieved by exposing the sensor to a series of known CO_2_ concentrations and recording the corresponding voltage response.

Controlled mixtures of CO_2_ and N_2_ gas (Airgas, certified ±5% accuracy) were introduced into the headspace of the inner chamber at concentrations of 50, 100, 200, 400, 600, 800, and 1000 ppm and allowed to equilibrate with deionized water (Milli-Q, 18.2 MΩ.cm) at the target temperature. The gas-liquid equilibrium is governed by Henry’s law (C_aq_ = K_H_.P_CO2_), which states that the dissolved CO_2_ concentration in water (C_aq_) is directly proportional to the partial pressure of CO_2_ in the headspace (P_CO2_), with K_H_ being the temperature-dependent Henry’s law constant [41]. By controlling the gas-phase composition and allowing sufficient equilibration time, a known and predictable dissolved CO_2_ concentration is thus established at the ATR crystal surface. For each concentration step, the system was allowed to stabilize for 120 s before recording the microbolometer output voltage, after the 30 min UV exposure. The full calibration sequence was repeated three times on separate days n = 3 independent replicates) to assess inter-day reproducibility.

To establish the measurement noise floor, the output voltage was recorded in the absence of IR radiation, yielding a baseline of 0.2 μV at ambient temperature. Noise floor characterization was performed using 3 consecutive data points under pure N_2_ purge at each test temperature. The limit of detection (LOD) was defined as 3σ/S, where σ is the standard deviation of the noise floor and S is the calibration sensitivity (μV/ppm), following IUPAC recommendations [42] and consistent with the LOD definitions used by the commercial instruments compared in Section 4.1. The noise floor σ was determined from n = 3 consecutive measurements under pure N_2_ purge at each temperature over approximately 5 min, and represents the post-lock-in noise level after rejection of 1/f and low-frequency drift components by phase-sensitive detection at 37 Hz, well above the microbolometer’s 1/f corner frequency (~1–5 Hz [29]). At ambient temperature, the resulting LOD was 35 ppm. We note that this represents a short-term LOD under controlled laboratory conditions. Establishing the LOD over deployment-relevant timescales (hours to days) would require additional characterization including Allan variance analysis and power spectral density measurements to quantify residual drift from sources such as LED spectral aging, ATR crystal surface contamination, and ambient temperature cycling; this is identified as an essential objective for future field-validation work.

Because the target application includes deployment in cold, deep-water environments where enhanced CO_2_ solubility and reduced detector noise may improve sensitivity, the full calibration and noise characterization were repeated at 5.0 ± 0.5 °C. Low-temperature conditions were maintained using a Galaxy CF5 Commercial Chest Freezer from WebstaurantStore, Salt Lake City, UT in USA, with temperature continuously monitored by a Fluke 62 Max Infrared Thermometer (±1.5 °C accuracy) bought from Fluke Electronics, Everett, WA in USA. The system was characterized for linearity, sensitivity, and signal-to-noise ratio (SNR) at both ambient and cold temperatures, enabling a direct comparison of sensor performance across the anticipated deployment range.

It should be noted that the gas inlet is used only during calibration. In operational field deployment, the sensor directly measures dissolved CO_2_ already present in the water body using the voltage-concentration transfer function established by this procedure. A non-zero dissolved CO_2_ baseline may exist before UV illumination due to: (i) ambient atmospheric CO_2_ (~420 ppm) dissolving during sample preparation (~15–20 ppm at equilibrium, 20 °C); (ii) slow aerobic decomposition of dissolved organic matter (DOM) in compost-amended samples; (iii) residual dissolved CO_2_ from prior measurements. All UV-induced CO_2_ values reported in this work represent the net increase above this established baseline.

### 2.3. UV-Induced CO_2_ Production Experiments

Beyond passive measurement of dissolved CO_2_, the sensor system was also used to quantify CO_2_ generated by UV-induced photochemical oxidation of organic matter in soil-water mixtures. This dual capability, combining ATR detection with UV stimulation, enables rapid estimation of labile soil organic carbon content. The following subsections describe the UV irradiation setup, the environmental parameters varied, and the data acquisition protocol.

#### 2.3.1. UV Irradiation Setup

Soil-water mixtures (mud) were individually subjected to UV-A (365 nm peak, 315–400 nm band, ~5 mW optical power), UV-B (305 nm peak, 280–315 nm band, ~3 mW), and UV-C (254 nm germicidal, ~4 mW) irradiation for 30 min per exposure. UV LEDs were positioned approximately 2 cm above the sample surface to ensure uniform illumination. To maintain experimental consistency, a fresh sample was prepared for each UV measurement, and the light source was changed between conditions. Replicate measurements (n = 3) were performed for each UV band to verify reproducibility.

#### 2.3.2. Environmental Parameter Variation

To systematically investigate how environmental conditions influence UV-induced CO_2_ generation, the following parameters were varied one at a time while holding all others constant:

pH: Solutions at pH 5–11 were prepared by adjusting the soil-water mixture with NaOH/HCl, validated using a digital MEXYBE pH meter (±0.1 pH units).

Temperature: Experiments were conducted at 5 °C, 10 °C, 20 °C, 25 °C, and 35 °C under thermoelectric temperature control.

Pressure: The enclosed system was pressurized to 1.0, 1.25, and 1.5 ATM by regulated N_2_ pressurization, with pressure verified by a calibrated gauge.

Organic substrate (compost): True Organic Berry Food 5-4-4 (OMRI certified) compost was added at varying concentrations (%DOC ranging from 2.7% to 21.6%) to simulate natural organic matter availability.

#### 2.3.3. Data Acquisition and Analysis

For each experimental condition, CO_2_ concentrations were continuously monitored by both the microbolometer-based ATR system and the commercial PASCO reference sensor throughout the UV exposure period. It should be noted that both the PASCO reference sensor and the microbolometer measure dissolved CO_2_. The PASCO sensor (PS-3208) from Pasco, Roseville, CA in USA employs a waterproof ePTFE (expanded polytetrafluoroethylene) membrane sleeve through which dissolved CO_2_ diffuses to an internal NDIR detector (manufacturer-specified equilibration time: ~3 min), while the microbolometer measures dissolved CO_2_ directly via ATR evanescent wave interaction at the crystal-liquid interface. No mechanical stirring was used. All measurements were recorded under a standardized fixed-duration protocol: 120 s of stabilization following gas introduction or UV exposure, during which dissolved CO_2_ accumulates in the aqueous phase through first-order mass-transfer dynamics. Because the release, conversion, and partitioning dynamics are linear and the exposure time is held constant across all calibration points, the integrated microbolometer response remains linearly proportional to the CO_2_ source concentration even when full gas-liquid equilibrium is not strictly achieved. The microbolometer signal was recorded every 30 s via lock-in amplification. Following the 30-min UV exposure, the UV lamp was turned off and the IR source/microbolometer system was activated to measure the resulting dissolved CO_2_ levels. Each experimental run followed a standardized sequence: noise floor establishment under pure N_2_, IR exposure and signal stabilization monitoring (over 120 s), comparison of the measured signal against room-condition baseline (~450 ppm atmospheric CO_2_), and SNR analysis. Signal-to-noise ratios were computed as the ratio of the stabilized CO_2_ signal to the baseline noise floor.

## 3. Results

This section presents the experimental results following the measurement workflow used to evaluate the microbolometer-based ATR CO_2_ sensor. We first establish the baseline sensor performance through calibration against a commercial reference instrument (Section 3.1). We then identify the optimal UV wavelength for stimulating CO_2_ production from soil-water mixtures (Section 3.2). After selecting the irradiation source, the sensor performance is examined across environmentally relevant temperature and pH conditions (Section 3.3). The influence of dissolved organic carbon (DOC) concentration is then evaluated (Section 3.4), followed by an assessment of pressure and UV wavelength effects across environmental conditions (Section 3.5). Finally, a consolidated statistical evaluation of system reliability is presented (Section 3.6).

### 3.1. Sensor Calibration and Baseline Performance

Prior to environmental testing, the microbolometer-based ATR sensor was calibrated against a commercial PASCO wireless CO_2_ sensor (PS-3208, accuracy ± 25 ppm + 5% of reading), which served as the reference instrument for establishing known CO_2_ concentrations. Controlled mixtures of CO_2_ and N_2_ were introduced into the chamber headspace at concentrations ranging from approximately 50 ppm to 1000 ppm, and the resulting microbolometer voltage response was recorded.

**Figure 3:**
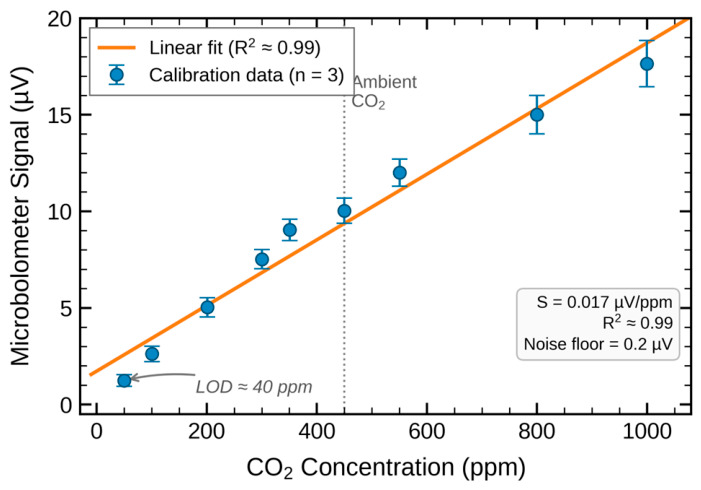
Calibration curve of the microbolometer-based ATR CO_2_ sensor at 25 °C, showing microbolometer output voltage (μV) versus PASCO-measured CO_2_ concentration (ppm) from 0 to 1000 ppm. Linear fit: R^2^ ≈ 0.99, sensitivity ≈ 0.017 μV/ppm. Error bars represent ±1 standard deviation from n = 3 independent replicate calibration runs. The strong linearity confirms Beer-Lambert law behavior across the tested range.

The calibration results are shown in Figure 3. The microbolometer output exhibited a highly linear response across the tested concentration range. The sensor signals increased from approximately 1.2 µV at 50 ppm to ~16.8 µV at 1000 ppm, demonstrating a clear monotonic relationship between CO_2_ concentration and microbolometer response.

Linear regression of the calibration data yielded an approximate sensitivity of ~0.017 µV ppm^−1^ with an excellent coefficient of determination (R^2^ ≈ 0.99), indicating strong linearity over the full measurement range. The error bars shown in Figure 3 represent the standard deviation from repeated measurements and remained small relative to the signal amplitude, confirming good measurement stability.

The baseline noise level of the system was approximately 0.2 µV, determined from repeated measurements under pure N_2_ purge conditions. At 450 ppm (near ambient atmospheric CO_2_), the microbolometer produced a signal of approximately 8 µV, corresponding to a signal-to-noise ratio (SNR) of approximately 40. At the lowest calibrated concentration of ~50 ppm, the signal of approximately 1.2 µV yields SNR ≈ 6, representing the lower-bound detection performance. Based on the 3σ/S criterion (Section 2.2), the practical limit of detection is approximately 35 ppm at 25 °C, consistent with the LOD values reported in Table 1.

Results throughout this study are presented in both reconstructed signal voltage (μV) and calibrated CO_2_ concentration (ppm) to provide complementary information. The voltage representation preserves the direct experimental observation after spectral reconstruction, enabling independent assessment of signal-to-noise ratio, sensitivity, and detection limits. The concentration representation, derived via the linear calibration transfer function (0.017 μV/ppm, R^2^ ≈ 0.99), facilitates direct comparison with the PASCO reference sensor and with environmental CO_2_ levels reported in the literature. The strong linearity of the calibration ensures that these two representations are interchangeable over the tested range (50–1000 ppm).

The calibration results confirm that the microbolometer sensor provides stable, linear, and repeatable detection of CO_2_ concentration over nearly one order of magnitude. Because the global calibration sensitivity (0.017 μV/ppm) was established at 25 °C, applying it directly at other temperatures introduced a systematic overestimation of 15–20% in the microbolometer-derived concentrations relative to the PASCO reference. This offset arises from the temperature dependence of both the evanescent wave penetration depth and the refractive index contrast at the ZnSe-water interface, which alter the effective optical path length at different temperatures. To account for this, temperature-specific linear recalibrations were performed by regressing PASCO reference concentrations against globally calibrated microbolometer values using n = 14 paired measurements per temperature pooled across 0% and 2% DOC conditions (5 °C: slope = 0.862, intercept = −14.0 ppm, R^2^ = 0.98; 10 °C: slope = 0.886, intercept = −5.8 ppm, R^2^ = 0.99; 25 °C: slope = 0.837, intercept = −8.5 ppm, R^2^ = 0.96). After recalibration, corrected accuracy exceeded 94% at all temperatures for concentrations above twice the limit of detection, with mean residual errors of 6.4% at 5 °C, 1.8% at 10 °C, and 3.1% at 25 °C. All accuracy values reported in subsequent sections refer to recalibrated measurements unless otherwise noted. Note that the calibration was performed under controlled laboratory conditions at 25 °C, additional experiments were conducted to evaluate sensor performance under varying environmental parameters, including temperature, pH, dissolved organic carbon concentration, pressure, and UV irradiation wavelength.

### 3.2. UV Wavelength Dependence of CO_2_ Production

To determine the optimal irradiation source for stimulating CO_2_ generation from soil-water mixtures, experiments were performed using three UV wavelength bands: UV-A (365 nm), UV-B (305 nm), and UV-C (254 nm). Soil-water mixtures were exposed to each wavelength for 30 min, followed by an additional 30-min observation period to monitor recovery dynamics. CO_2_ concentration was continuously recorded using the PASCO reference sensor. It should be noted that all microbolometer ATR measurements were performed after the 30-min UV exposure period was complete, not during active irradiation. Furthermore, full gas-liquid thermodynamic equilibrium is not a prerequisite for the validity of the sensor comparison, because both the PASCO reference sensor (which samples dissolved CO_2_ through its ePTFE membrane) and the microbolometer (which samples dissolved CO_2_ via ATR evanescent absorption) measure the same dissolved-phase quantity through independent physical mechanisms under an identical fixed-duration protocol. Their correlation therefore reflects a genuine measurement agreement independent of the absolute gas-liquid partitioning state.

**Figure 4:**
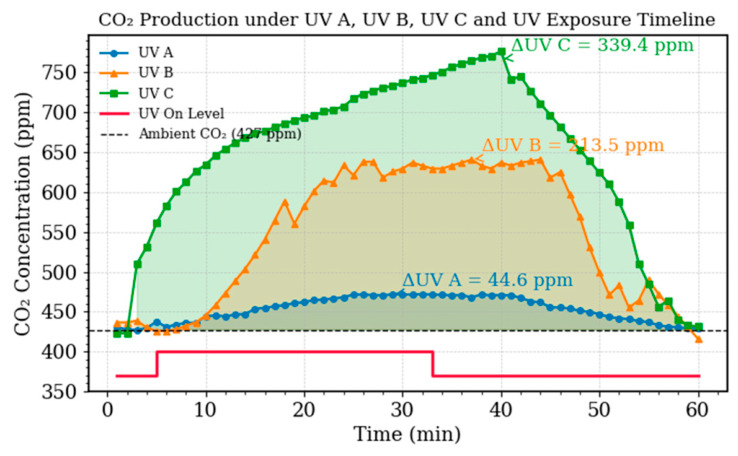
CO_2_ concentration (ppm) over time under UV-A (blue circles), UV-B (orange triangles), and UV-C (green squares) irradiation of soil-water mixtures. Red bar indicates UV-on period (5–35 min). Dashed line: ambient CO_2_ baseline (~427 ppm). Shaded regions represent net CO_2_ production above baseline: ΔUV-C = 339.4 ppm, ΔUV-B = 213.5 ppm, ΔUV-A = 44.6 ppm. After UV-off (t > 35 min), CO_2_ concentrations declined toward baseline as generated CO_2_ degassed from the open system.

The resulting time-resolved CO_2_ profiles are shown in Figure 4. All experiments began near the ambient baseline of approximately 427 ppm. Upon UV activation, CO_2_ concentrations increased rapidly, with the magnitude of production strongly dependent on wavelength. UV-C irradiation produced the most pronounced response, reaching ΔCO_2_ ≈ 339 ppm above baseline within approximately 20 min. UV-B generated a moderate increase of ΔCO_2_ ≈ 213 ppm, while UV-A produced only a weak enhancement of ΔCO_2_ ≈ 45 ppm, close to the sensor detection threshold.

The observed hierarchy (UV-C ≫ UV-B > UV-A) is consistent with the photon energy required to break organic bonds. UV-C photons at 254 nm carry approximately 4.88 eV, exceeding the dissociation energies of both C-C (3.6 eV) and C-O (3.7 eV) bonds and enabling direct photolytic fragmentation of organic molecules into CO_2_ and smaller products. In contrast, UV-B photons provide borderline energy for direct cleavage and instead promote a combination of photolysis and reactive oxygen species (ROS) formation via chromophoric dissolved organic matter (CDOM). UV-A photons lack sufficient energy for direct bond rupture and primarily drive indirect ROS-mediated oxidation pathways.

Temporal profiles further support these mechanisms. UV-C produced a rapid initial CO_2_ burst (~25 ppm min^−1^) followed by gradual saturation, indicating rapid consumption of labile organic substrates. UV-B exhibited a slower but sustained increase (~7 ppm min^−1^), consistent with ROS-mediated oxidation processes. UV-A showed the slowest response, reflecting its reliance on indirect photochemical pathways.

Based on these results, UV-C was selected as the irradiation source for all subsequent experiments, as it consistently produced CO_2_ concentrations well above the sensor detection limit within the experimental measurement window.

### 3.3. Temperature-Dependent Sensor Performance

To evaluate the performance of the microbolometer-based ATR sensor under varying environmental conditions, measurements were conducted at three temperatures (5 °C, 10 °C, and 25 °C) while simultaneously varying the solution pH between 5 and 11. Soil-water mixtures without added dissolved organic carbon (DOC = 0%) were used to isolate CO_2_ production originating from native soil carbon under UV-C irradiation. The pH of the solution was adjusted using dilute HCl and NaOH to avoid introducing additional inorganic carbon.

**Figure 5:**
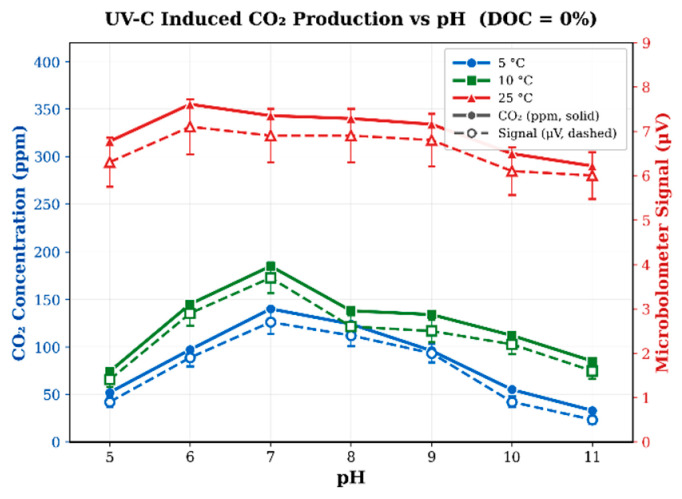
Temperature-dependent sensor performance under UV-C irradiation (30 min, 0% DOC) vs. pH (5–11). Right axis: reconstructed CO_2_ absorption signal at 4.26 μm (μV, red); left axis: PASCO-measured CO_2_ concentration (ppm, blue). Error bars represent ±1 SD (n = 3).

Figure 5 shows the measured CO_2_ concentrations obtained from the PASCO reference sensor together with the corresponding microbolometer voltage signal measured under identical conditions. The PASCO measurements (solid lines) represent the CO_2_ concentration in ppm, while the dashed curves represent the corresponding microbolometer signal response. The close correspondence between these measurements demonstrates that the microbolometer sensor accurately tracks variations in dissolved CO_2_ across both temperature and pH conditions.

At 25 °C, the highest CO_2_ production was observed, with PASCO-measured concentrations reaching approximately 355 ppm near pH ≈ 6, corresponding to a peak microbolometer signal of approximately 7.1 μV. Using the global calibration sensitivity of 0.017 μV/ppm established in Section 3.1, this signal corresponds to a microbolometer-derived concentration of approximately 418 ppm. The systematic offset between the PASCO measurement and the globally calibrated microbolometer-derived concentration (15–20% across temperatures) arises primarily from applying a single calibration sensitivity established at 25 °C across all temperatures. The evanescent wave penetration depth (Equation (1)) depends on the refractive indices of both the ZnSe crystal and the aqueous medium, both of which vary with temperature, resulting in a temperature-dependent effective optical path length not captured by a fixed calibration slope. After applying the temperature-specific linear recalibration described in Section 3.1, corrected measurement accuracy exceeded 94% across all tested temperatures for concentrations above twice the LOD. As the pH increased toward alkaline conditions, the measured CO_2_ concentration gradually decreased, consistent with the shift in dissolved inorganic carbon equilibrium toward bicarbonate and carbonate species.

At 10 °C, the PASCO-measured peak CO_2_ concentration was approximately 185 ppm near pH ≈ 7, corresponding to a microbolometer signal of approximately 3.7 μV. Using global calibration, this voltage corresponds to a microbolometer-derived concentration of approximately 218 ppm. Similarly, at 5 °C the PASCO-measured peak CO_2_ concentration was approximately 140 ppm near pH ≈ 7, corresponding to a microbolometer signal of approximately 2.7 μV, which converts to approximately 159 ppm using the same calibration relationship.

Although the magnitude of CO_2_ production decreases with decreasing temperature, the microbolometer response consistently follows the same trend as the PASCO measurements across the entire pH range. This agreement, observed under post-UV fixed-exposure conditions where both sensors independently sample dissolved-phase CO_2_, indicates that the microbolometer consistently tracks relative changes in dissolved CO_2_ across the tested temperature and pH range. We note that this comparison was performed under controlled laboratory conditions with a standardized measurement protocol; further evaluation under fully dynamic non-equilibrium conditions encountered in natural environments would be needed to confirm sensor performance during active CO_2_ flux events.

The sensor performance metrics extracted from these measurements are summarized in Table 1. In this study, CO_2_ concentrations derived from the microbolometer signal were calculated using the global calibration sensitivity of 0.017 μV/ppm obtained from the calibration curve in Figure 3. While the calibration slope is constant across temperatures, the effective detection limit varies slightly with temperature due to changes in the measured noise floor. Based on the measured noise levels (Table 1), the estimated limits of detection are approximately 26 ppm at 5 °C, 30 ppm at 10 °C, and 35 ppm at 25 °C. The improved detection capability at lower temperatures is attributed to decreased thermal infrared background emission from the colder water sample, which reduces the stochastic photon noise incident on the microbolometer and lowers the effective noise floor, increasing the signal-to-noise ratio even though the intrinsic sensitivity of the device remains unchanged.

Across all temperatures and pH values, the uncorrected microbolometer-derived concentrations showed a systematic overestimation of 15–20% relative to the PASCO reference due to the use of a single global calibration. This offset arises from the temperature dependence of the evanescent wave penetration depth (Equation (1)): changes in water temperature alter the refractive index contrast at the ZnSe-water interface, modifying the effective optical path length through the sample. After applying temperature-specific linear recalibration, residual errors were reduced to 1.8–6.4% on average, with Pearson correlation coefficients of r ≥ 0.98 at all three temperatures. The high linearity of the recalibration (R^2^ ≥ 0.96) confirms that the offset is systematic, reproducible, and fully correctable, consistent with an optical-path origin rather than detector instability. Although the outer chamber temperature was not independently logged, it is thermally buffered from the inner chamber by an air gap and continuous room-temperature N_2_ purging, maintaining the detector environment near ambient laboratory conditions (~22 °C). The lower noise floor observed at reduced sample temperatures (0.15 µV at 5 °C vs. 0.20 µV at 25 °C) is attributed to decreased thermal infrared background emission from the colder water sample rather than to cooling of the detector element itself. Because the microbolometer is a broadband thermal detector, a colder sample produces less stochastic IR background radiation along the optical path, resulting in a cleaner baseline independent of the detector’s own temperature.

### 3.4. Effect of Dissolved Organic Carbon on CO_2_ Production

#### 3.4.1. Effect of 2% DOC Across Temperatures and pH

With the sensor’s temperature-dependent baseline established using 0% DOC samples (Section 3.3), we next examine how dissolved organic carbon modifies UV-induced CO_2_ production across the full temperature and pH range.

**Figure 6:**
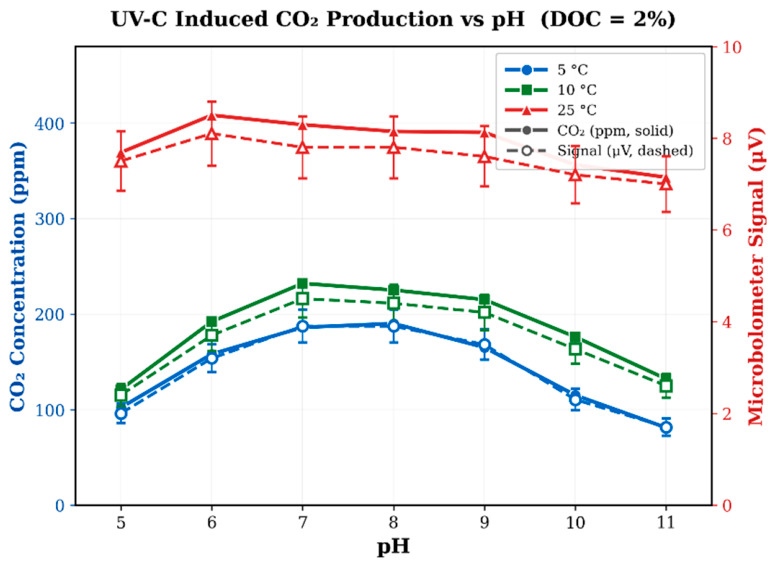
Effect of 2% DOC on UV-C-induced CO_2_ production vs. pH (5–11) at (a) 5 °C, (b) 10 °C, (c) 25 °C, compared with 0% DOC in Figure 5. Reconstructed CO_2_ signal (μV) shown on right axis. Error bars: ±1 SD (n = 3).

Figure 6 presents the results with 2% DOC compost added to the soil-water mixtures. Comparing these results with the 0% DOC measurements in Figure 5 reveal consistent increases in CO_2_ production while preserving the bell-shaped pH profile observed previously.

At 5 °C, peak CO_2_ increased from approximately 159 ppm to 229 ppm (+44%; signal 2.7→3.9 μV). At 10 °C, the increase was approximately 218→265 ppm (+22%; signal 3.7→4.5 μV). At 25 °C, the enhancement was more modest, increasing from approximately 418 ppm to 476 ppm (+14%; signal 7.1→8.1 μV). CO_2_ concentrations reported here were calculated from the measured microbolometer voltage using the global calibration sensitivity of 0.017 μV/ppm established in Section 3.1. It should be noted that these values represent dissolved-phase CO_2_ only. Because CO_2_ solubility decreases with temperature [41], the dissolved-phase concentrations at higher temperatures underestimate total CO_2_ production relative to lower temperatures, where a greater fraction of generated CO_2_ remains in solution.

These results demonstrate that the addition of dissolved organic carbon increases UV-induced CO_2_ production across all tested temperatures while maintaining the same pH dependence observed in the DOC-free measurements. Importantly, the intrinsic sensor parameters (noise floor, sensitivity, and accuracy) remained unchanged between the 0% and 2% DOC experiments, confirming that DOC primarily affects the CO_2_ generation chemistry rather than the microbolometer measurement capability.

The increasing relative DOC enhancement at lower temperatures (44% at 5 °C compared with 14% at 25 °C) is an observation that may be explained by several factors. The most straightforward explanation is temperature-dependent CO_2_ solubility: the Henry’s law constant K_H_ increases by approximately 39% from 25 to 5 °C [41], so a substantially larger fraction of photochemically generated CO_2_ remains in the dissolved phase, where the ATR sensor measures at lower temperatures, amplifying the apparent DOC enhancement. Temperature-dependent changes in photochemical quantum yields and reaction kinetics may also contribute, though quantifying these effects would require dedicated actinometry experiments. Although UV-C (254 nm) is germicidal, sterilization is dose-dependent rather than instantaneous; bacterial endospores and organisms shielded within soil aggregates can survive significant UV-C exposure [43], and UV radiation may elicit complex biological responses beyond simple inactivation in certain microorganisms [44]. The photochemical generation of reactive oxygen species (ROS) under UV-C irradiation provides a well-documented abiotic pathway for organic matter oxidation to CO_2_ [45], which is consistent with the observed UV wavelength hierarchy (UV-C >> UV-B > UV-A). However, no sterile controls, autoclaved blanks, or metabolic inhibitor experiments were performed in this study; therefore, a definitive mechanistic attribution is not possible from the present data. Future studies incorporating these controls would be needed to quantitatively separate photochemical, solubility-driven, and any residual biological contributions. From a practical perspective, the observation that the DOC-induced CO_2_ enhancement is proportionally larger at lower temperatures indicates that the soil organic carbon (SOC) assay capability of the sensor is most sensitive under colder conditions, which is advantageous for the intended deep-water and cold-environment deployment applications.

#### 3.4.2. DOC Concentration Series

**Figure 7:**
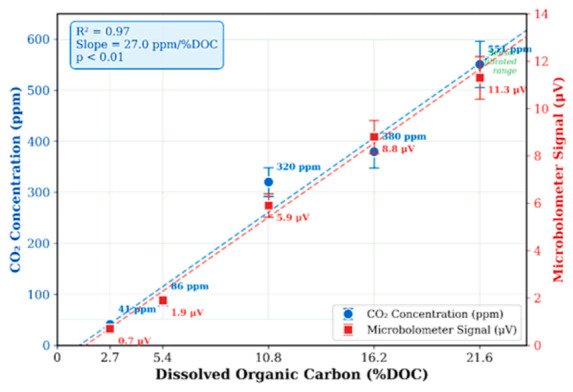
DOC vs. CO_2_ production at 25 °C. Left y-axis: CO_2_ (ppm); right y-axis: reconstructed CO_2_ absorption signal (μV). R^2^ = 0.97. Error bars: ±1 SD (n = 3).

To further investigate the relationship between dissolved organic carbon concentration and UV-induced CO_2_ production, a systematic DOC concentration series was conducted at 25 °C under UV-C irradiation for 30 min. The results are shown in Figure 7. As DOC concentration increased from 2.7% to 21.6%, the microbolometer signal increased from 0.7 μV to 11.3 μV. Using the global calibration relationship (0.017 μV/ppm), this corresponds to CO_2_ concentrations increasing from approximately 41 ppm to 665 ppm.

The relationship between DOC concentration and CO_2_ production exhibited strong linearity (R^2^ = 0.97, *p* < 0.01), indicating that dissolved organic carbon serves as a primary substrate for photochemical CO_2_ generation under UV-C irradiation over the tested concentration range. No saturation behavior was observed up to 21.6% DOC, suggesting that the photochemical conversion capacity was not exhausted under these conditions; however, confirming true rate-limiting behavior would require testing at higher DOC concentrations to identify the onset of kinetic saturation. The DOC range examined (2.7–21.6%) extends beyond typical mineral soil organic carbon levels (0.5–5%) but is representative of organic-rich marine sediments and coastal benthic environments, where dissolved organic carbon can comprise well over 20% of pore water organic matter [46]. The lower end of this range (2.7–5.4%) is most relevant to typical environmental conditions, while the higher concentrations characterize the sensor’s full dynamic range. This linear dependence demonstrates the potential of the microbolometer-based ATR sensor to serve not only as a dissolved CO_2_ detector but also as a tool for quantitative estimation of soil organic carbon through UV-stimulated photochemical conversion.

### 3.5. Effects of Pressure and UV Wavelength on CO_2_ Production

After characterizing the effects of temperature, pH, and dissolved organic carbon on the microbolometer response in the previous sections, we next investigated how UV wavelength, hydrostatic pressure, and temperature influence UV-induced CO_2_ production. These parameters are particularly relevant for evaluating sensor performance under realistic environmental conditions where sunlight spectrum, water depth, and ambient temperature may vary.

**Figure 8:**
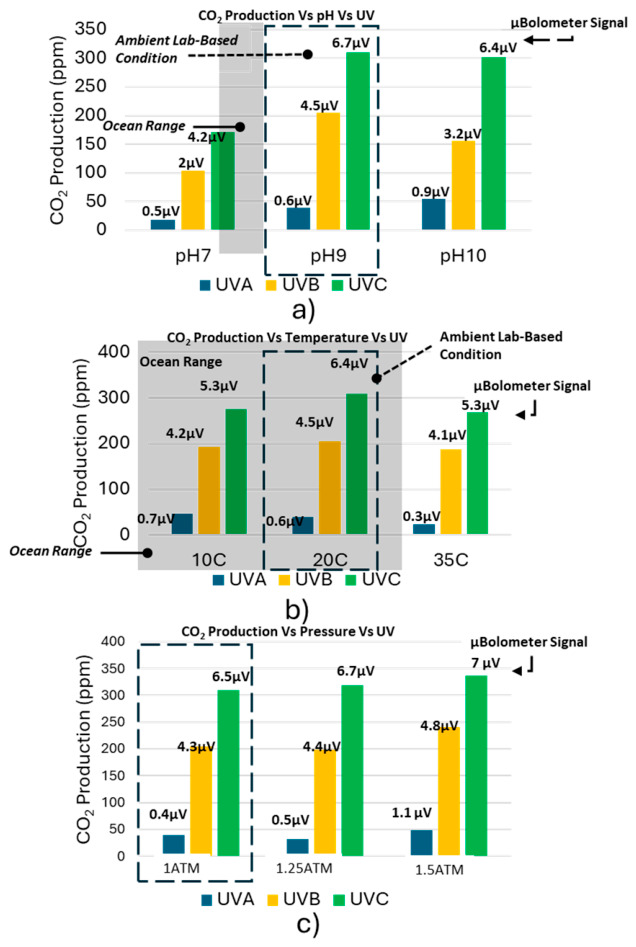
UV-induced CO_2_ production and microbolometer signal under UV-A (blue), UV-B (yellow), and UV-C (green) across environmental conditions. Top left: CO_2_ vs. pH (ambient lab). (**a**) CO_2_ vs. temperature. (**b**) CO_2_ vs. pressure (1.0, 1.25, 1.5 ATM). (**c**) CO_2_ vs. pH. Reconstructed CO_2_ absorption signal values (μV) annotated. UV-C > UV-B > UV-A under all conditions.

Figure 8 summarizes CO_2_ production and the corresponding microbolometer signal under three UV wavelengths (UV-A, UV-B, and UV-C) while varying temperature, pressure, and pH. In contrast to the temperature range used in Section 3.3 (5–25 °C), which was selected to represent typical environmental conditions for sensor performance evaluation, the temperatures used in Figure 8 (10, 20, and 35 °C) were chosen to examine how thermal conditions influence UV-induced photochemical CO_2_ generation. This extended temperature range enables clearer observation of temperature-dependent photochemical processes independent of the pH-dependent trends described earlier.

Across all experimental conditions, a consistent UV wavelength hierarchy was observed. UV-C irradiation produced the highest CO_2_ concentrations and the strongest microbolometer signals, followed by UV-B, while UV-A generated only weak signals close to the noise floor. From the temperature series (10–35 °C) shown in Figure 8, the microbolometer signals under UV-A were very small (≈ 0.3–0.7 µV), UV-B produced intermediate signals (≈ 4.1–4.5 µV) and UV-C generated the largest signals (≈ 5.3–6.4 µV). This hierarchy is consistent with the photon energy of the UV wavelengths. UV-C radiation at 254 nm (≈4.88 eV) exceeds the dissociation energies of typical organic bonds such as C-C (3.6 eV) and C-O (3.7 eV), enabling direct photolytic cleavage of organic molecules and enhanced CO_2_ production.

The influence of hydrostatic pressure on CO_2_ production was also examined across the range 1.0–1.5 ATM, corresponding approximately to 0–5 m water depth in shallow coastal environments. Under UV-C illumination, the microbolometer signal increased slightly with pressure, producing approximately 6.5 μV at 1.0 ATM, 6.7 μV at 1.25 ATM, and 7.0 μV at 1.5 ATM. This modest increase is consistent with Henry’s law, where higher pressure increases the solubility and retention of dissolved CO_2_ in the aqueous phase.

Figure 8 also compares the microbolometer response across several pH conditions. Although the pH range examined in this figure differs from the more detailed pH sweep presented in Section 3.3, the results remain consistent with the earlier observations that the microbolometer signal reliably tracks the relative changes in CO_2_ production. The sensor response remains strongest under UV-C illumination across all tested pH values.

Importantly, the microbolometer signal consistently followed the same trends observed in the PASCO reference CO_2_ measurements across all UV wavelengths, temperatures, pressures, and pH conditions. This agreement confirms that the microbolometer response reflects actual variations in dissolved CO_2_ concentration rather than artifacts arising from changes in illumination conditions or environmental parameters.

Overall, these results demonstrate that the microbolometer-based ATR sensor maintains stable and reliable CO_2_ detection across a broad range of environmental variables. The ability of the sensor to accurately track CO_2_ variations under different UV wavelengths, temperatures, and pressures further validates its suitability for environmental monitoring applications in shallow aquatic and soil-water systems.

### 3.6. System Reliability and Statistical Summary

To evaluate the overall reliability of the measurement platform, temperature-specific linear recalibration was performed at each temperature using n = 14 paired measurements (microbolometer-derived vs. PASCO reference CO_2_ concentration) pooled across 0% and 2% DOC conditions and spanning pH 5–11. The recalibration parameters are summarized in Table 1. Prior to recalibration, the microbolometer systematically overestimated CO_2_ concentrations by 15–20% relative to the PASCO reference when using the global calibration sensitivity of 0.017 μV/ppm established at 25 °C. This offset is attributed to the temperature dependence of the evanescent wave penetration depth and the refractive index contrast at the crystal-water interface. After applying temperature-specific linear recalibration, corrected accuracy exceeded 94% at all temperatures for concentrations above twice the limit of detection, with mean residual errors of 6.4% at 5 °C, 1.8% at 10 °C, and 3.1% at 25 °C. Pearson correlation coefficients between the recalibrated microbolometer and PASCO measurements were r = 0.99 at 5 °C, r = 0.99 at 10 °C, and r = 0.98 at 25 °C. Triplicate relative standard deviations remained below 5% across all conditions and typically 2–3% at concentrations above 200 ppm. Residual analysis of the corrected data showed that 95% of measurement deviations fell within ±8% of the reference values.

Collectively, these results demonstrate that the microbolometer-based ATR sensor provides robust and statistically reliable dissolved CO_2_ measurements across the full range of environmental variables tested, including temperature (5–25 °C), pH (5–11), pressure (1.0–1.5 ATM), and dissolved organic carbon concentrations up to 21.6%. The systematic nature of the uncorrected offset, fully explained by temperature-dependent optical path variation and correctable through a linear recalibration at each temperature, does not limit the practical utility of the sensor for environmental monitoring applications where temperature is concurrently measured.

## 4. Discussion

### 4.1. Comparative Analysis with Commercial Sensors

The developed microbolometer-based ATR sensor offers several distinct advantages over existing commercial underwater CO_2_ sensors. Table 2 provides a detailed comparison. Note that some parameters for our system reflect the current laboratory prototype; values in parentheses indicate target/projected performance not yet demonstrated in the field.

The key advantages of the microbolometer ATR approach include: (1) the ability to directly measure dissolved CO_2_ in the liquid phase through ATR evanescent wave interaction, eliminating the rate-limiting membrane diffusion step of conventional submersible sensors; (2) extremely low power consumption in the milliwatt range, compatible with battery-powered autonomous platforms; (3) MEMS-compatible fabrication enabling low-cost mass production; (4) the unique capability to simultaneously serve as both a CO_2_ detector and a soil organic carbon analyzer when combined with UV illumination.

LOD context and target applications: The current LOD (26–35 ppm) is higher than the sub-μatm sensitivity of dedicated oceanographic pCO_2_ instruments. However, the target applications do not require sub-ppm sensitivity: sediment pore waters (500–5000 ppm CO_2_), SOC assay (40–550 ppm dynamic range demonstrated), hydrothermal vent plumes (>1000 ppm), and coastal benthic flux chambers (>100 ppm). For open-ocean dissolved CO_2_ monitoring where sub-μatm precision is required, commercial membrane-NDIR sensors remain more appropriate. Our system fills a complementary niche: low-cost, rapid, direct measurement in high-concentration near-sediment environments. The LOD can potentially be improved through longer optical path lengths, multi-pass ATR configurations, or signal averaging.

### 4.2. Implications for In Situ Environmental Monitoring

The combined microbolometer ATR + UV photo-oxidation system enables a novel class of environmental sensors: a “sediment carbon scanner” that can be deployed in ocean sediment or soils to rapidly estimate total organic carbon content in situ. By illuminating a sediment sample with UV and measuring the resulting CO_2_ increase, one can infer the labile organic carbon content without laboratory analysis, providing results in minutes rather than days. Our findings on pH, temperature, and pressure effects can guide the optimization of such field sensors. The strong DOC-CO_2_ correlation (R^2^ = 0.97) obtained under controlled laboratory conditions demonstrates the feasibility of this approach, although independent field validation with natural soil and sediment samples remains necessary to confirm its applicability in real-world environments.

For deployment at shallow to moderate depths where temperature is low and pressure is elevated, the ATR approach offers a unique advantage: it can detect dissolved CO_2_ directly without requiring headspace extraction. While the signal strength is reduced under cold, high-pressure conditions, the sensor maintained detectable response even at 5 °C, and strategies such as longer UV exposure, local heating, or agitation could enhance the signal. The demonstrated dual-function capability, real-time CO_2_ monitoring combined with rapid SOC estimation, is unavailable in any existing commercial system. An important caveat when interpreting the temperature-dependent CO_2_ data (Section 3.3, Table 1) is that all reported concentrations represent the dissolved phase only, as measured at the ATR crystal surface. CO_2_ solubility in water decreases with increasing temperature, the Henry’s law constant K_H_ decreases by approximately 39% from 5 to 25 °C [41], meaning that a larger fraction of photochemically generated CO_2_ partitions into the headspace at higher temperatures and is not captured by the dissolved-phase measurement. Consequently, the total CO_2_ production (dissolved + gas-phase) at 25 °C is likely underestimated relative to 5 °C, and the apparent temperature trends in Table 1 do not fully represent the true enhancement in photochemical CO_2_ generation rate with temperature. No solubility correction was applied, because the primary function of the sensor is to quantify dissolved CO_2_ directly, the quantity that governs carbonate equilibria, pH, and biological processes in the water column. For applications requiring total carbon budget calculations, a solubility correction incorporating concurrent temperature and pressure measurements would be necessary. Future field-deployable versions will integrate temperature-dependent calibration models to account for these partitioning effects.

### 4.3. Challenges and Limitations

Several challenges must be addressed before transitioning from the current laboratory prototype to field deployment. It is important to emphasize that all results presented in this study were obtained exclusively under controlled laboratory conditions using prepared aqueous solutions and commercially sourced compost. The sensor has not been tested in any natural water body, and no field performance data is currently available. The limitations described below should therefore be understood as anticipated challenges requiring future experimental characterization, rather than resolved engineering constraints. In particular, in situ deployment introduces challenges that are not presented in a laboratory setting: ensuring waterproof integrity and pressure tolerance for all submerged electronics, managing power budgets for long-term autonomous operation, and addressing measurement noise arising from continuously changing temperature, pH, and dissolved gas solubility in natural water bodies. Performance under realistic field conditions, including variable salinity, turbidity, biofouling, and temperature fluctuations, remains to be validated. A submersible titanium housing with waterproof IR-transparent windows and a gas-permeable membrane is currently under development, with structural integrity calculations confirming pressure tolerance exceeding 100 m depth.

Among the environmental factors not fully characterized in this study, salinity and turbidity are particularly relevant for marine deployment. Salinity affects dissolved CO_2_ through the salting-out effect, reducing Henry’s law solubility by approximately 3–4% per 10 PSU [41]; this represents a systematic and correctable offset. Turbidity introduces potential optical scattering interference, although the extremely short evanescent wave penetration depth (~1.0–1.5 μm) provides inherent robustness against bulk particulate scattering, since only molecules within the near-surface evanescent field contribute to the absorption signal. Systematic characterization across 0–35 PSU salinity and 0–1000 NTU turbidity is planned for future work.

Long-term operational stability presents another important consideration. In this study, measurement repeatability was characterized through triplicate measurements (n = 3, separate days), yielding relative standard deviations below 5% across all conditions. However, individual experimental sessions lasted 1–3 h, and no dedicated continuous-operation stability test was performed over extended periods (e.g., 24+ hours at a fixed CO_2_ concentration). Autonomous field deployment would require unattended operation for weeks to months (>720 h), representing a stability gap of more than one order of magnitude beyond what has been demonstrated. A dedicated Allan variance or drift analysis over deployment-relevant timescales remains an essential step for future field validation. Potential drift sources include microbolometer responsivity changes, LED spectral and intensity aging, and ATR crystal surface degradation. Closely related is the challenge of biofouling: organic and biological film accumulation on the ATR crystal surface in marine environments would progressively attenuate the evanescent wave interaction and degrade measurement accuracy. Planned mitigation strategies include copper-alloy sensor frames to inhibit biofilm growth, mechanical wipers for periodic surface cleaning, and UV-C self-cleaning pulses that leverage the existing UV illumination subsystem.

Finally, cross-sensitivity to other dissolved species warrants further investigation. While the computational spectral reconstruction algorithm targets the 4.26 μm CO_2_ absorption band and inherently provides spectral discrimination by resolving the absorption spectrum across the full 3–5 μm band, potential interferents present in natural waters, including dissolved methane (3.3 μm), hydrogen sulfide, and colored dissolved organic matter, have not been systematically tested. Similarly, the pressure range tested in this study was limited to 1.0–1.5 ATM, corresponding to approximately 0–5 m water depth. Extending operation to deep-sea environments (>100 m, >11 ATM) will require the pressure-rated housing currently under development. Dark controls, autoclaved blanks, and sodium azide-inhibited samples are planned to definitively partition UV-induced CO_2_ between photolytic, photochemical oxidative, and biological sources. Empirical validation in complex natural matrices across the full range of anticipated deployment conditions remains essential before the system can be considered field ready.

## 5. Conclusions

This study presented the development and characterization of a microbolometer-based ATR mid-infrared sensor for direct dissolved CO_2_ detection in aquatic and sediment environments, combined with UV-induced photochemical CO_2_ generation for rapid soil organic carbon assessment. To the best of our knowledge, this represents the first integration of MEMS microbolometer detectors with ATR evanescent-wave spectroscopy for direct liquid-phase dissolved CO_2_ measurement, eliminating the membrane equilibration step required by all existing commercial underwater CO_2_ sensors.

The sensor demonstrated excellent linearity (R^2^ ≈ 0.99) over a 50–1000 ppm detection range, with a practical limit of detection of approximately 26 ppm at 5 °C, an improvement attributed to the combined effects of enhanced CO_2_ solubility, reduced detector noise, and improved thermal stability at lower temperatures. Among the UV wavelengths investigated for photochemical CO_2_ generation from soil-water mixtures, UV-C (254 nm) proved most effective, producing a net increase of 339 ± 17 ppm above baseline within 30 min consistent with direct photolytic cleavage of organic bonds, although abiotic ROS-mediated pathways may also contribute. Across all tested environmental conditions, temperatures of 5–35 °C, pH 5–11, and pressures of 1.0–1.5 ATM, the sensor achieved corrected accuracies of 94–98% relative to a commercial reference instrument (Pearson r ≥ 0.98) after applying temperature-specific linear recalibration to account for the temperature dependence of the evanescent wave optical path length.

While the current system remains a laboratory prototype and has not been tested in natural water bodies, the demonstrated dual-function capability, simultaneous real-time dissolved CO_2_ monitoring and rapid sediment organic carbon estimation within a single compact platform, addresses a measurement gap not currently filled by existing commercial instruments. Several significant challenges remain before field deployment can be realized, including: (i) validation of performance under realistic conditions of variable salinity (0–35 PSU), turbidity, and biofouling; (ii) demonstration of long-term operational stability beyond the triplicate-measurement reproducibility tested here; (iii) development and pressure-testing of a waterproof submersible housing with reliable sealing for all electronic subsystems; (iv) miniaturization of the lock-in amplifier and data acquisition electronics to meet the power and size constraints of autonomous platforms; (v) calibration against natural soil and sediment samples of known organic carbon content. Future efforts will systematically address each of these challenges.

## Figures and Tables

**Table 1:** Summary of temperature-dependent sensor performance parameters (0% DOC, UV-C, 30 min).

Parameter	5 °C	10 °C	25 °C
LOD (ppm)	~26	~30	~35
Sensitivity (μV/ppm) (calibration)	0.017	0.017	0.017
Recalibration Slope	0.862	0.886	0.837
Recalibration intercept (ppm)	−14.0	−5.8	−8.5
Recalibration R^2^	0.98	0.99	0.96
Uncorrected accuracy at peak (%)	~86	~82	~82
Accuracy (%)	~94 (>2 × LOD)	~98	~97
Peak CO_2_ (ppm) (PASCO)	~140 (pH 7)	~185 (pH 7)	~355 (pH 6)
Peak CO_2_ (ppm) (bolometer derived)	159	~218	~418
Peak reconstructed signal (μV)	2.7	3.7	7.1
Noise floor (μV)	~0.15	~0.17	~0.20

**Table 2:** Comparison of the developed microbolometer ATR sensor with commercial underwater CO_2_ sensors. Values in parentheses indicate projected (not yet demonstrated) performance.

Parameter	This Work	Pro-Oceanus CO_2_-Pro CV [17]	CONTROS HydroC [18]	Bench-top ATR-FTIR [24,25]
Detection Range	50–1000+ ppm	0–2000 μatm	0–6000 μatm	~10–1000+ ppm
LOD(demonstrated)	26–35 ppm	<1 μatm (~0.4 ppm)	±1% FS	~10 ppm
Response Time	~3 s (stabilization)	~60 s (membrane equation)	~60 s (membrane equation)	<5 s
Power	<50 mW (demonstrated) *	~1 W	~1.5 W	>50 W
Size	~15 cm (lab prototype)	>30 cm (field unit)	>40 cm (field unit)	Bench-top
Cost (est.)	<$500 (components) **	$15,000-$20,000	>$20,000	>$50,000
Measurement Phase	Direct liquid	Gas (membrane equation)	Gas (membrane equation)	Direct liquid
Cooling Required	No	No	No	Yes (LN_2_)
SOC Assay Function	Yes (demonstrated)	No	No	No
Depth Rating	(Target: 100 m, untested)	6000 m (rated)	6000 m (rated)	Lab only
Field Deployments	Lab only (current)	Multiple campaigns	Year+ autonomous	Lab only

* (Core sensing module: microbolometer + IR LED + lock-in amplifier during active measurement). Full system peak power during measurement cycle: ~1.3 W (including UV LED, wireless communication). Under daily duty-cycling (measurement cycle ~5 min/day, standby ~23.9 hr/day), average power consumption is ~1–2 mW. ** Commercial sensor prices ($15,000–$50,000+) reflect complete field-ready systems including pressure housings, calibration, firmware, and manufacturer support. Our figure represents an early-stage laboratory prototype.

## Data Availability

The raw data supporting the conclusions of this article will be made available by the authors on request.
